# Changes in Blood microRNA Expression and Early Metabolic Responsiveness 21 Days Following Bariatric Surgery

**DOI:** 10.3389/fendo.2018.00773

**Published:** 2019-01-04

**Authors:** Stephen L. Atkin, Vimal Ramachandran, Noha A. Yousri, Manasi Benurwar, Steven C. Simper, Rodrick McKinlay, Ted D. Adams, S. Hani Najafi-Shoushtari, Steven C. Hunt

**Affiliations:** ^1^Department of Medicine, Weill Cornell Medicine, Doha, Qatar; ^2^Department of Cell and Developmental Biology, Weill Cornell Medicine, Doha, Qatar; ^3^MicroRNA Core Laboratory, Weill Cornell Medicine, Doha, Qatar; ^4^Department of Genetic Medicine, Weill Cornell Medicine, Doha, Qatar; ^5^Rocky Mountain Associated Physicians, Inc., Salt Lake City, UT, United States; ^6^Department of Internal Medicine, University of Utah, Salt Lake City, UT, United States; ^7^Intermountain LiveWell Center, Intermountain Healthcare, Salt Lake City, UT, United States

**Keywords:** microRNA, bariatric surgery, gastric bypass, biomarkers, diabetes

## Abstract

**Background:** Early metabolic responses following bariatric surgery appear greater than expected given the initial weight loss and coincide with improvement in diabetes. We hypothesized that small non-coding microRNA changes might contribute to regulating mechanisms for metabolic changes and weight loss in patients with severe obesity and diabetes.

**Methods:** Twenty-nine type 2 patients with severe obesity (mean BMI 46.2 kg/m^2^) and diabetes underwent Roux-en-Y gastric bypass (RYGB) surgery. Clinical measurements and fasting blood samples were taken preoperatively and at day 21 postoperatively. Normalization of fasting glucose and HbA1c following bariatric surgery (short-term diabetes remission) was defined as withdrawal of anti-diabetic medication and fasting glucose < 100 mg/dL (5.6 mmol/L) or HbA1c < 6.0%. MicroRNA expression was determined by quantitative polymerase chain reaction and tested for significant changes after surgery.

**Results:** BMI decreased by 3.8 kg/m^2^ 21 days postoperatively. Eighteen of 29 RYGB (62%) had short-term diabetes remission. Changes from pre- to post-surgery in 32 of 175 microRNAs were nominally significant (*p* < 0.05). Following multiple comparison adjustment, changes in seven microRNAs remained significant: miR-7-5p, let-7f-5p, miR-15b-5p, let-7i-5p, miR-320c, miR-205-5p, and miR-335-5p. Four pathways were over-represented by these seven microRNAs, including diabetes and insulin resistance pathways.

**Conclusion:** Seven microRNAs showed significant changes 21 days after bariatric surgery. Functional pathways of the altered microRNAs were associated with diabetes-, pituitary-, and liver-related disease, with expression in natural killer cells, and pivotal intestinal pathology suggesting possible mechanistic roles in early diabetes responses following bariatric surgery.

## Perspective Section

- Metabolic changes following bariatric surgery can be rapid and precede substantial weight loss, the causes of which remains unclear.
- Non-coding microRNAs were measured before and 21 days after bariatric surgery and showed changes associated with bariatric surgery.
- The physiological pathways associated with these microRNAs may identify biomarkers of surgical response and an understanding of weight loss or long-term diabetes remission.

## Introduction

Obesity has become a worldwide epidemic and continues to increase in prevalence. For the severely obese, bariatric surgery has been established as the most effective intervention leading to significant and sustained weight loss and improving systemic metabolism to control the co-morbidities associated with severe obesity. Bariatric surgery has resulted in improvements in health-related quality of life, longevity, remission of type 2 diabetes, and reduction in long-term cancer risk (1–6). One perplexing aspect seen in patients with type 2 diabetes is the rapid improvement in glycemic control long before rapid weight loss ensues. The mechanisms through which bariatric surgery leads to these beneficial results are multifactorial and have been reported to include the BRAVE effects (bile flow alteration, reduction of gastric size, anatomical gut rearrangement and altered flow of nutrients, vagal manipulation, and enteric gut hormone modulation), improvement of the gut microbiome, and weight-related physiological and disease-modifying effects (7, 8). However, the translation of these physiologic effects into direct clinical benefits remains poorly understood. Furthermore, the identification of bariatric surgical patients who fail to lose much weight or regain most of their weight lost remains challenging to predict. Being able to identify non-responders before surgery would be of particular importance for patients with obesity or diabetes and considering bariatric surgery.

MicroRNAs are endogenous, non-coding RNAs regulating gene expression at the post-transcriptional level. These RNA transcripts are ~22 nucleotides in length and bind to target messenger RNA (mRNA) transcripts either entirely or partially, leading to mRNA cleavage, translational repression, and mRNA decay (9–12). Each mRNA may bind to multiple base pairing so enabling each of the many distinct human microRNA sequences to affect many target mRNA expressions regulating extensive biological and cellular pathways. MicroRNAs, may therefore control virtually every biological process. It is, therefore, not surprising that microRNAs have been associated with obesity and its resulting co-morbidities (13). MicroRNAs have been shown to regulate adipogenesis, insulin secretion, glucose uptake, lipid metabolism, and other biological pathways related to obesity (14–17).

MicroRNAs act at the intracellular level but they are also found in the circulation, complexed with protein (particularly Argonaute) or within exosomes (18). The mechanisms through which microRNAs move into the circulation and the functional implications of microRNA expression in the circulation are unknown. In their protein-complexed or exosomal forms, these small RNA molecules are stable in the circulation, are not readily digested by ribonucleases and can withstand repeated freeze-thaw cycles, thus allowing for long-term storage without compromising microRNA integrity (19). The speed and sensitivity of microRNA expression through quantitative PCR and other profiling platforms, facilitates the use of microRNAs as novel biomarkers having therapeutic potential (20, 21). Circulating microRNAs have been proposed as biomarkers for cancer, tissue damage, heart failure, and polycystic ovary syndrome (20, 22, 23). A circulating microRNA signature in obesity has also been recently described (13), and diabetes has been associated with a distinct circulating microRNA profile (19).

Because fasting glucose levels often normalize after bariatric surgical procedures but before significant weight loss, this study investigated whether or not patients with pre-surgical type 2 diabetes had changes in microRNAs immediately following bariatric surgery. The aim of this analysis was to use state-of-the-art microRNA analysis to determine potential biomarkers of early metabolic and diabetes response 21 days following surgery.

## Materials and Methods

### Subject Selection

From February 1, 2010, to June 16, 2011, patients seeking bariatric surgery at the Rocky Mountain Associated Physicians surgical center, Salt Lake City, were recruited for participation in this study. Twenty-nine severely obese (BMI≥35 kg/m^2^), diabetic patients gave written informed consent to participate and had Roux-en-Y gastric bypass (RYGB) surgery. Surgeries were performed by three surgeons (partners) using the same surgical techniques. This study was approved by the University of Utah's Institutional Review Board.

Selection criteria were ages 25–60, either gender, all races, BMI≥35 kg/m^2^ to < 60 kg/m^2^, no active cancer, no history of myocardial infarction, coronary bypass surgery, PTCA, or stroke, and confirmed the presence of treated type 2 diabetes, defined by current use of diabetes prescription medications. All subjects were severely obese, sedentary, and not taking regular aerobic exercise.

### Testing Protocol

Each patient was examined at the University of Utah Cardiovascular Genetics Division research clinic a week before bariatric surgery, at their home 7 days post-surgery, and at the research clinic 21 days following surgery. Patients are asked to consume a high protein, low-calorie (≤ 1,000 calories) diet 2 weeks prior to their surgery. Because diabetes is often remitted within days following surgery and with minimal weight loss, 21-days was selected for the final post-surgery exam to test for weight-independent changes in microRNAs since surgery. At each examination, subjects' weight, waist circumference, hip circumference, percent body fat and lean mass (bioelectrical impedance) were measured. Also, following a 5-min rest, three sitting blood pressures were obtained 1 min apart. The average of the last two blood pressures was used for analysis. Height, basic demographic and medical history questionnaires were collected at baseline only. A spot urine sample for microalbumin and creatinine was obtained at baseline. Prior to each examination, participants were asked to fast for a 12-h period. Lipids were measured by ultracentrifugation (24). Clinical chemistries and HbA1c were measured at each exam. Diabetes remission following bariatric surgery was defined as withdrawal of all anti-diabetic medication, fasting glucose < 126 mg/dL (7 mmol/L), and HbA1c < 6.5% at the 21-day examination.

### MicroRNA Analysis

Currently, due to accuracy, simplicity, and greater reproducibility, quantitative polymerase chain reaction (qPCR) is the favored method for determining microRNA expression when compared to other hybridization or sequencing-based technologies (25). microRNA was isolated from 200μL plasma using the miRCURY™ RNA Isolation Kit (Exiqon A/S, Denmark) following the manufacturer's instructions and applying an RNA Spike-in kit (Exiqon A/S, Denmark) for assessment of hemolysis and contamination with blood cells and RNA isolation efficiency. The yield of microRNA preparation from plasma samples was monitored by including carrier RNA from the bacteriophage MS2. The quality and the integrity of the RNA were assessed by a 2100 Bioanalyzer instrument (Agilent Technologies), equipped with the small RNA specific chip. The expression of microRNA levels was retrieved from a microRNA data set generated by RNA sequencing of more than 390 different human tissue and primary cell samples (26).

### Statistics

Mean normalization was performed before analysis using the global mean of each microRNA. Using GenEx software that accompanied the Bioanalyzer, normalization was performed to achieve a global mean of all microRNAs with Ct below 35. A paired *t*-test was used to test the paired changes from pre-surgery to 21 days post-surgery in microRNA levels. General linear models for the change in microRNA were also used to adjust for gender, age, and pre-surgery BMI. Change in BMI was added to a model to test if the change in microRNA was due to change in BMI. Finally, microRNA changes were also compared between those whose glucose or HbA1c normalized after 21 days by adding a dichotomous variable for short-term diabetes remission to the general model. False discovery rates (FDR) (27) of *q* < 0.1 were considered significant enough to carry forward to pathway analysis. Pathway analysis was performed using Ingenuity Pathway Analysis (Qiagen). Those pathways over-represented at *p* < 1.0 × 10^−6^ by the FDR-significant microRNA changes were identified along with the microRNA-associated diseases for those pathways.

## Results

Table 1 shows the description of the 29 subjects enrolled in this study. The group BMI mean was 46.2 kg/m^2^, and 69% of patients were female. BMI decreased by 3.8 kg/m^2^ during the 21 days after surgery. Eighteen (62%) of the 29 subjects (6 males and 12 females) had normalization of levels of fasting glucose or HbA1c after 21 days. All oral hypoglycemic agents were stopped at discharge and insulin was titrated on discharge and follow-up.

**Table 1:** Study subject characteristics.

**Variable**	**Pre-surgery**	**Post-surgery**
% Female	69	69
Age (y)	47.5 ± 8.5	47.5 ± 8.5
BMI (kg/m^2^)	46.2 ± 9.9	42.4 ± 9.3^***^
Weight (kg)	129.6 ± 29.1	118.7 ± 26.9^***^
Waist circumference (cm)	135.2 ± 18.4	126.6 ± 19.3^***^
HbA1c (%)	7.1 ± 1.2^†^	6.3 ± 0.8^***^
Diabetes age at onset (y)	42.0 ± 8.1	*N*/*A*
Diabetes duration (y)	5.7 ± 5.4	*N*/*A*
Systolic blood pressure (mmHg)	122.2 ± 15.3	113.1 ± 18.1^**^
Diastolic blood pressure (mmHg)	72.0 ± 8.3	67.1 ± 7.8^**^
LDL cholesterol (mg/dL)	109.8 ± 33.9	98.5 ± 32.6
Triglycerides (mg/dL)	167.5 ± 52.7	138.8 ± 54.4^*^
HDL cholesterol (mg/dL)	42.0 ± 10.3	34.8 ± 6.9^***^

*Mean ± SD; N = 29*.

* *p < 0.05*,

** *p < 0.01*,

*** *p < 0.001 vs. pre-surgery*.

† *One person on insulin only at baseline, 9 on both insulin and oral medications, and 19 on oral medications only*.

After quality control of the data, 175 microRNAs remained for analysis. Table 2 shows the paired *t*-test of the 21-day change in microRNA levels (post- minus pre-surgery). Thirty-two of the microRNA changes were nominally significant at the *p* < 0.05 level. After adjustment for multiple comparisons using a false discovery rate of *q* < 0.1, seven microRNA changes remained significant. Gender, baseline BMI and change in BMI were not significantly associated with any of the seven microRNA changes. Age was associated with microRNAs miR-15b-5p, miR-205-5p, and miR-335-5p (all *p* < 0.05). Adjustment of the microRNA changes for age made the microRNA changes even more significant than without adjustment.

**Table 2:** microRNA changes (post- minus pre-surgery).

**microRNA**	**Paired *t*-test**	***p*-value**	***q*-value**
hsa-miR-7-5p	5.01	1.35E-05	0.002
hsa-let-7f-5p	4.12	0.0003	0.026
hsa-miR-205-5p	3.53	0.0015	0.055
hsa-miR-15b-5p	3.47	0.0017	0.055
hsa-let-7i-5p	−3.47	0.0017	0.055
hsa-miR-320c	3.43	0.0019	0.055
hsa-miR-335-5p	3.32	0.0025	0.063
hsa-miR-148a-3p	3.03	0.0052	0.11
hsa-miR-502-3p	2.96	0.0062	0.11
hsa-miR-21-5p	2.95	0.0064	0.11
hsa-miR-186-5p	2.88	0.0075	0.12
hsa-miR-93-5p	−2.82	0.0088	0.12
hsa-miR-424-5p	2.79	0.0095	0.12
hsa-miR-25-3p	−2.78	0.0096	0.12
hsa-miR-652-3p	−2.69	0.012	0.13
hsa-miR-885-5p	2.67	0.013	0.13
hsa-miR-194-5p	2.65	0.013	0.13
hsa-miR-223-3p	−2.62	0.014	0.13
hsa-miR-16-5p	−2.60	0.015	0.13
hsa-miR-154-5p	2.58	0.016	0.14
hsa-miR-362-3p	2.44	0.021	0.18
hsa-miR-148b-3p	−2.39	0.024	0.19
hsa-miR-20b-5p	2.33	0.027	0.21
hsa-miR-106a-5p	−2.24	0.034	0.23
hsa-miR-185-5p	−2.24	0.033	0.23
hsa-let-7d-3p	−2.24	0.033	0.23
hsa-miR-19b-3p	−2.21	0.036	0.23
hsa-miR-1260a	2.15	0.040	0.24
hsa-miR-18a-5p	−2.14	0.041	0.24
hsa-miR-122-5p	2.14	0.041	0.24
hsa-miR-126-5p	2.05	0.049	0.27
hsa-miR-2110	2.05	0.049	0.27

**Figure 1:**
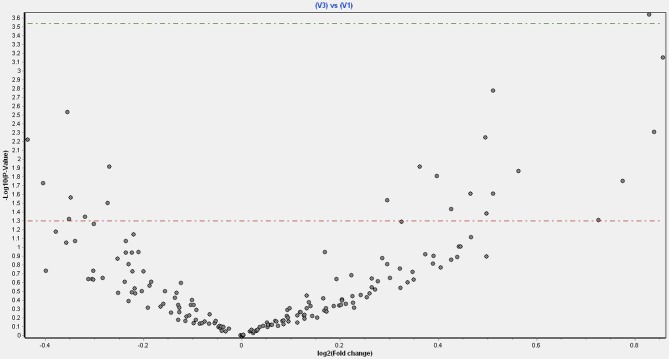
Volcano plot of fold changes in microRNA and significance level.

**Figure 2:**
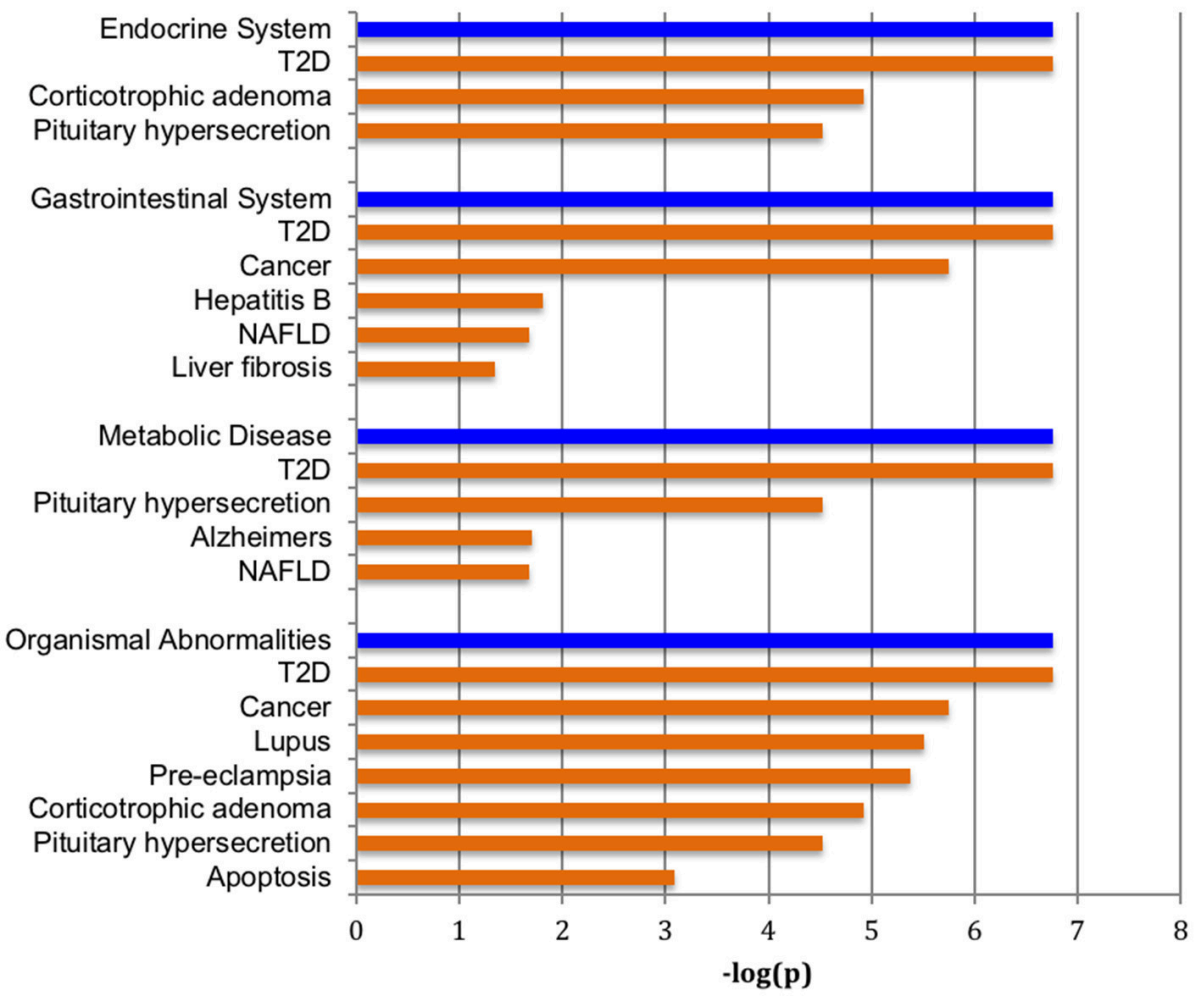
Pathways enriched with significantly changed microRNAs. Major over-represented pathways found to be associated with significantly altered set of microRNAs and weight change are highlighted in blue bars at *p* < 1.0 × 10^−6^. Diseases significantly correlated within the four significant pathways are shown in tan bars.

**Figure 3:**
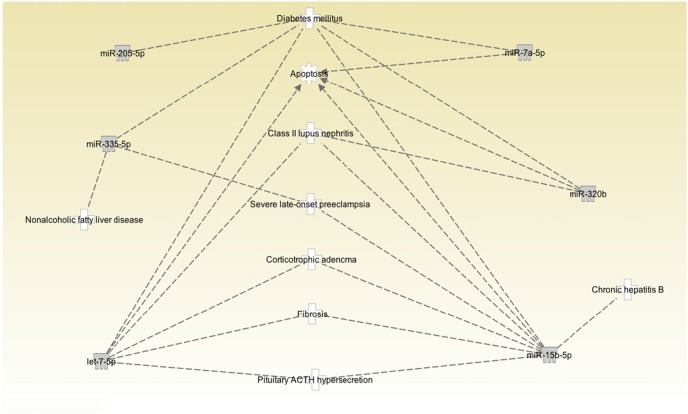
Pathway connections among the correlated diseases and significantly changed microRNAs. MicroRNAs let-7f-5p and let-7i-5p are in the same family and are labeled as let-7-5p.

Figure 1 shows a volcano plot of the log *p*-value vs. the log of microRNA fold change. Figure 2 shows the pathways and diseases within four of the most relevant pathways that were associated with the significant changes in the microRNAs after weight loss. Notably, the outcome shows among other disease related pathways, enrichment for metabolic diseases. Figure 3 shows the connections of the pathways from Ingenuity Pathway Analysis with the significant microRNAs.

There was one nominally significant beta coefficient (hsa-miR-132-3p; *p* < 0.05) from the regression of change in microRNA (post- minus pre-surgery) vs. normalization of levels of fasting glucose or HbA1c (yes vs. no). None of the microRNA changes had a false discovery rate *q* < 0.1.

**Figure 4:**
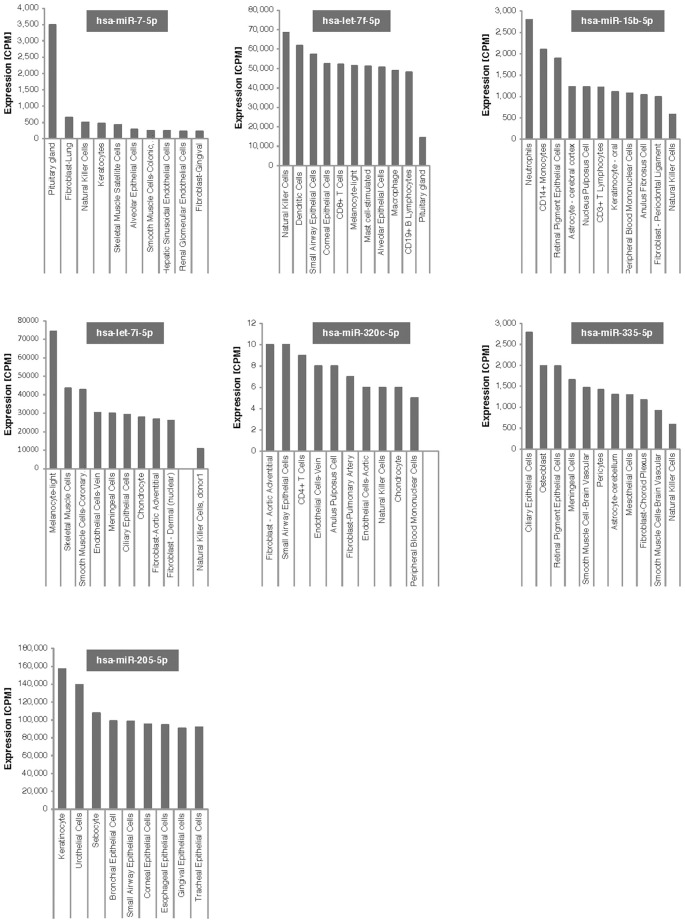
Tissue expression of the 7 microRNAs associated with weight change after bariatric surgery. Expression is given in counts per million reads (CPM).

As shown in Figure 4, six of the seven microRNAs that significantly changed after bariatric surgery were expressed in natural killer cells, although at different levels of expression. MicroRNA let7f-5p, which showed the 2nd largest increase after bariatric surgery, shows high expression levels in natural killer cells.

## Discussion

In post-surgical bariatric patients, the disparity between the rate of diabetes remission and the lagging rate of weight loss remains an area of intense clinical interest. In this small study we report that after adjustment for multiple comparisons, 7 microRNAs (miR-7-5p, let-7f-5p, miR-15b-5p, miR-7i-5p, miR-320c, miR-205-5p, and miR-335-5p) were significantly altered at 21 days post-surgery and with relatively minimal weight loss (3.8 kg).

Studies of bariatric surgery in diabetic obese patients support an immediate metabolic improvement, which is independent of weight loss and is likely due to dietary restrictions (28–32). However, there is little data on the changes in microRNA within a short time frame following a bariatric procedure. In a small study of 8 Chinese patients with Type 2 diabetes and with a high BMI (>30), changes in microRNA were seen when measured 3 months postoperatively, including miR-320c and the let-7 family, though no correlation could be made with diabetes remission (33). In another small study, all Type 2 diabetes patients (*n* = 12) no longer required insulin therapy at 12 months post-surgery, and miR-320a changed significantly (34). In a recent study on 9 non-diabetic patients undergoing RYGB surgery (35), microRNA measured at 1 month showed a significant decrease in mir-338-3p and an increase in miR-574-3p, neither of which were seen in this study, though at 3 months let-7b-5p was seen in both studies. A hyperinsulinemic-euglycemic clamp study showed a significant association of miR-16 (same genomic locus as 15b), miR-33, miR-107, miR-150, and miR-222 with insulin sensitivity (36). In a small study of six subjects, miR-16 decreased in adipose tissue 1 year after bariatric surgery and was associated with a decrease in insulin resistance (37).

Other studies have shown that 2 years after gastric bypass surgery, 72–79% of diabetes remitted (38, 39). Since only 62% of diabetics in this study showed normalization of levels of fasting glucose and HbA1c after 21 days, our study suggests that the length of follow-up requires more than 21 days to determine if diabetes remission has occurred. The choice of criteria to define remission of T2D after bariatric surgery is still widely debated. In agreement with the recommendations from an expert consensus meeting organized by the American Diabetes Association (40) partial remission of diabetes was defined as a fasting glucose level of 100 to 125 mg/dL and a HbA1c level of < 6.5% for at least 1 year without active pharmacologic therapy; complete remission was defined as a fasting glucose level of 100 mg/dL and a HbA1c level of < 6.0% for at least 1 year without active pharmacologic therapy; thus, it remains unclear if true diabetes remission had occurred in the patients in this study. The microRNA changes seen in this study with surgery itself are likely to be more informative and less confounded than the changes for diabetes-associated microRNAs.

Muscle is a major factor in peripheral insulin resistance, but the reported microRNAs related to insulin sensitivity in muscle (41) were not reflected in the microRNA changes shown here after only 21 days. This suggests that a change in peripheral insulin resistance may not be the major cause of acute diabetes remission and that the mechanism underlying diabetes remission may be at the level of the pancreas. It is known that knock out of Dicer in mice, critical in microRNA formation, leads to severe pancreatic dysfunction specifically at the level of the beta cell (42). A pancreatic explanation for early remission in diabetes may be inferred from a 12-year follow-up of patients following bariatric surgery in which diabetes patients on insulin, and therefore with potentially greater beta cell loss, showed the least remission compared with those on diet alone or on antidiabetes medication (6).

In obesity, there is a low-grade inflammatory response that reflects in activation of the body's immune system. This obesity-induced inflammation of visceral adipose tissue is likely to be an important contributor to insulin resistance and the movement of proinflammatory macrophages to the adipose tissue, conditions that can progress to type 2 diabetes (43). Therefore, if inflammation is reduced after bariatric surgery as adipose tissue is being lost, the large percentage of subjects showing a remission of diabetes is not surprising. A recent study of gastric bypass surgery found significant associations with reductions in multiple inflammatory markers, such as IL-6, CRP, ICAM-1, and t-PA:Ag, even though they were not correlated with weight loss (44).

Dysfunctional natural killer cells in tissue may be responsible for obesity-induced adipose stress and inflammation. Lowering the number of natural killer cells prevented infiltration of macrophages into visceral fat and reduced insulin sensitivity (43). High fat diets increase natural killer cells and proinflammatory cytokines (45). These studies suggest that short-term reduction in natural killer cells, through lower fat, post-bariatric surgery diets would reduce insulin resistance and reduce adipose tissue macrophages possibly through TNFα (45). Another study showed that although the percent of natural killer cells did not change, natural killer cell cytotoxic activity was improved 6 months after bariatric surgery (46). As shown in Figure 4, all but one of the microRNAs that showed significant changes at 21 days post-surgery are expressed in natural killer cells, with a number of them expressed at high levels, particularly let7f-5p. MicroRNA let7f-5p has the 2nd largest increase after bariatric surgery in our study and may be the best candidate for further follow-up studies. We don't know whether post-surgical microRNA changes seen at 21-days persist long-term or what their potential long-term effects on killer cells might be. Speculatively, short-term natural killer cell expression may be closely associated with bariatric surgery.

All seven of the microRNAs have direct pathways to diabetes, with subsets of the microRNAs related to other relevant pathways such as NAFLD and liver fibrosis, and pituitary function. MicroRNAs let-7f-5p and miR-7-5p show significant tissue expression in the pituitary and could contribute to the control of adrenal and thyroid hormone release. Bariatric surgery affected 11β-hydroxysteroid dehydrogenase activity, reflecting a possible reduction in hypothalamic pituitary adrenal axis drive, while TSH levels decreased and free thyroxine levels increased (47, 48). Liver fibrosis and non-alcoholic liver disease are also included in two of the pathways, both of which improve after bariatric surgery. Significant changes in the expression of circulating microRNAs could also potentially reflect major ongoing physiological changes in cells such as apoptosis despite the minimal weight loss seen in this study. As weight is eventually lost, extra adipose cells are removed from the tissues.

Of those microRNAs changing within 21 days of surgery, miR-7 shows the most significant increase. A recent study shows that miR-7 is implicated in regulation of pancreatic beta cell function in mouse and human islet cells (49). Notably, miR-7a expression levels are decreased in obese/diabetic mouse models and human islets from obese and moderately diabetic individuals with compensated β cell function. This could also be reflected in a decreased amount of miR-7 in circulation. Consequently, and in accordance to our observation in this study, weight loss and improved insulin sensitivity could restore the relatively higher level of miR-7 as shown in Table 2.

MiR-7-5p has been demonstrated to decrease the expression level of trefoil factor 3 (TFF3) that is involved in reconstructing the epithelial barrier by stimulating epithelial migration and proliferation (50). In addition to the inhibition of TFF3, miR-7-5p inhibits LS174T cell proliferation and migration via inhibiting the PI3K/Akt signaling pathway (50). miR-7f-5p remains an unknown quantity with little data on its biological role, though it has been shown to be upregulated with some other microRNA in colorectal cancer(51), and may, therefore, have an intestinal endothelial role to play. MiR let-7i-5p has been shown to be involved in murine and human down-regulation and inhibition of beige adipocyte function, thus inhibiting the stimulation of brown thermogenic adipose tissue (52). This may suggest that bariatric surgery facilitates brown thermogenic fat accumulation that would add to the potential weight loss seen. The miR-320 family, including miR-320c, targets SOX4, FOXM, and FOXQ1 (53). Notably, increased expression of miR-320c has been shown to enhance adipocyte differentiation and accelerate the formation in mature adipocytes *in-vitro* (54). Recently it has been shown that miR-320 transfection inhibited AdipoR1 protein levels that inhibited downstream adiponectin levels (55) in an animal model, whilst global upregulation of miR-320 expression in duodenal-jejunal bypass in these animals resulted in impaired gluconeogenesis, lipid metabolism, and increased inflammatory markers (55). miR-205-5 has been associated with multiple conditions that may be linked through proliferative states such as colorectal (56), prostate (57), and breast cancer (58) and, therefore, has a key role in cellular proliferation, including that of the GI tract. Similarly, miR-335-5p is associated with differing proliferative processes in bone, but its down-regulation in several organs, including stomach and pancreatic cancer, has been reported (59).

Limitations of the study include the small sample size of subjects, particularly the number who had diabetes remission. This makes it more difficult to assess overlap between the microRNA involvement in obesity and diabetes. Also, we did not test function of the microRNAs, but only association. The best predictors of diabetes remission are lower baseline glycemia and shorter diabetes duration (60), and indeed there is no direct evidence demonstrating that BMI is a predictor of diabetes remission after bariatric surgery.

Not surprisingly, these results suggest that several independent processes associated with bariatric surgery and weight loss are coinciding, and are not necessarily the same mechanisms related to metabolic features such as normalization of levels of fasting glucose and HbA1c. Several microRNAs showed significant changes within 21 days after bariatric surgery. The altered microRNAs could be related to decreases in general or adipose-specific cellular proliferation, apoptosis and natural killer cells, and changes in intestinal cell biology and pathology. If these microRNAs are found in larger studies or longer term follow-up studies to be related to diabetes remission, that may suggest similar mechanistic roles as for obesity, through natural killer cells, reduction in insulin resistance and inflammation leading to the early the diabetes response following bariatric surgery.

## Author Contributions

SS, RM, TA, and SH were involved in undertaking the study. VR, SN-S, and MB were involved in sample analysis and data analysis. SA, SH, and NY were involved in data analysis. All authors were involved in the preparation of the manuscript and approved of its submission.

### Conflict of Interest Statement

The authors declare that the research was conducted in the absence of any commercial or financial relationships that could be construed as a potential conflict of interest.
